# Regulation of Mutant p53 Protein Expression

**DOI:** 10.3389/fonc.2015.00284

**Published:** 2015-12-17

**Authors:** Reshma Vijayakumaran, Kah Hin Tan, Panimaya Jeffreena Miranda, Sue Haupt, Ygal Haupt

**Affiliations:** ^1^Tumour Suppression Laboratory, Peter MacCallum Cancer Centre, Melbourne, VIC, Australia; ^2^Sir Peter MacCallum Department of Oncology and Department of Pathology, The University of Melbourne, Parkville, VIC, Australia; ^3^Department of Biochemistry and Molecular Biology, Monash University, Clayton, VIC, Australia

**Keywords:** mutant p53, Mdm2, miRNA, proteasomal degradation, cancer

## Abstract

For several decades, p53 has been detected in cancer biopsies by virtue of its high protein expression level which is considered indicative of mutation. Surprisingly, however, mouse genetic studies revealed that mutant p53 is inherently labile, similar to its wild type (wt) counterpart. Consistently, in response to stress conditions, both wt and mutant p53 accumulate in cells. While wt p53 returns to basal level following recovery from stress, mutant p53 remains stable. In part, this can be explained in mutant p53-expressing cells by the lack of an auto-regulatory loop with Mdm2 and other negative regulators, which are pivotal for wt p53 regulation. Further, additional protective mechanisms are acquired by mutant p53, largely mediated by the co-chaperones and their paralogs, the stress-induced heat shock proteins. Consequently, mutant p53 is accumulated in cancer cells in response to chronic stress and this accumulation is critical for its oncogenic gain of functions (GOF). Building on the extensive knowledge regarding wt p53, the regulation of mutant p53 is unraveling. In this review, we describe the current understanding on the major levels at which mutant p53 is regulated. These include the regulation of p53 protein levels by microRNA and by enzymes controlling p53 proteasomal degradation.

## Introduction

Wild type (wt) p53 is a tumor suppressor, which plays a key role in the cellular stress response. Abrogating p53 function is a key event in human cancer, leading to deregulated cell cycle, genomic instability, resistance to stress signals, and ultimately leading to cancer development (1, 2). Dysfunction of p53 occurs in half the cases of cancers by direct mutations in the gene, whereas in the remainder, p53 becomes dysfunctional through a variety of regulatory breakdowns. Mutant p53 fails to emulate the transcriptional program executed by wt p53 to provide a robust response to stress.

Most p53 mutations are missense (hotspot mutations – R175, G245, R248, R249, R273, R282) and occur at its DNA-binding domain, which accounts for the improper DNA engagement and disruption of transcriptional activity. P53 mutants also gain new oncogenic functions ­including resistance to chemotherapies, enhanced cell growth, metabolism, and invasion [reviewed in Ref. (3, 4)].

Surprisingly, mutant p53, like its wt counterpart, is inherently labile (5–7). Sustained degradation of wt p53 in healthy cells protects them against potent cell growth inhibition, while stress provokes p53 accumulation and activation (8). Similarly, mutant p53 accumulates in response to stress (7). Thus, both wt and mutant p53 need to accumulate in order to execute their respective functions: wt p53 suppresses cancer while mutant p53 promotes cancer through its GOFs. It is therefore of great clinical importance to understand how wt and mutant p53 are regulated if we are to tailor treatments according to p53 status. That is, either reactivating wt p53 expression and function or counteracting mutant p53. In this review, we will outline the major levels at which mutant p53 is regulated and discuss the major players. As most of the regulation appears to occur post-transcriptionally [reviewed in Ref. (9)], this will form the major focus of this review.

## Regulation of Mutant p53 by microRNA

MicroRNAs (miRNA) are the best-characterized members of the non-coding RNAs (ncRNAs) family. Typically these are 18–24 nucleotide (nt) RNA molecules that are not translated into proteins, and target messenger RNA (mRNA) species, through engagement of as few as six complementary nucleotides (10). Canonically, mature miRNAs bind mRNA 3′-untranslated-regions (3′-UTRs) and promote either target degradation or translational inhibition. Through engagement of coding regions (11), 5′-UTR (12), and open reading frames (ORF) (13), miRNAs can also regulate translation. The targeting flexibility of miRNAs allows them to affect multiple targets that are pertinent to both tumor suppression and oncogenesis: gene expression, protein regulation, homeostasis, and diseases.

**Figure 1:**
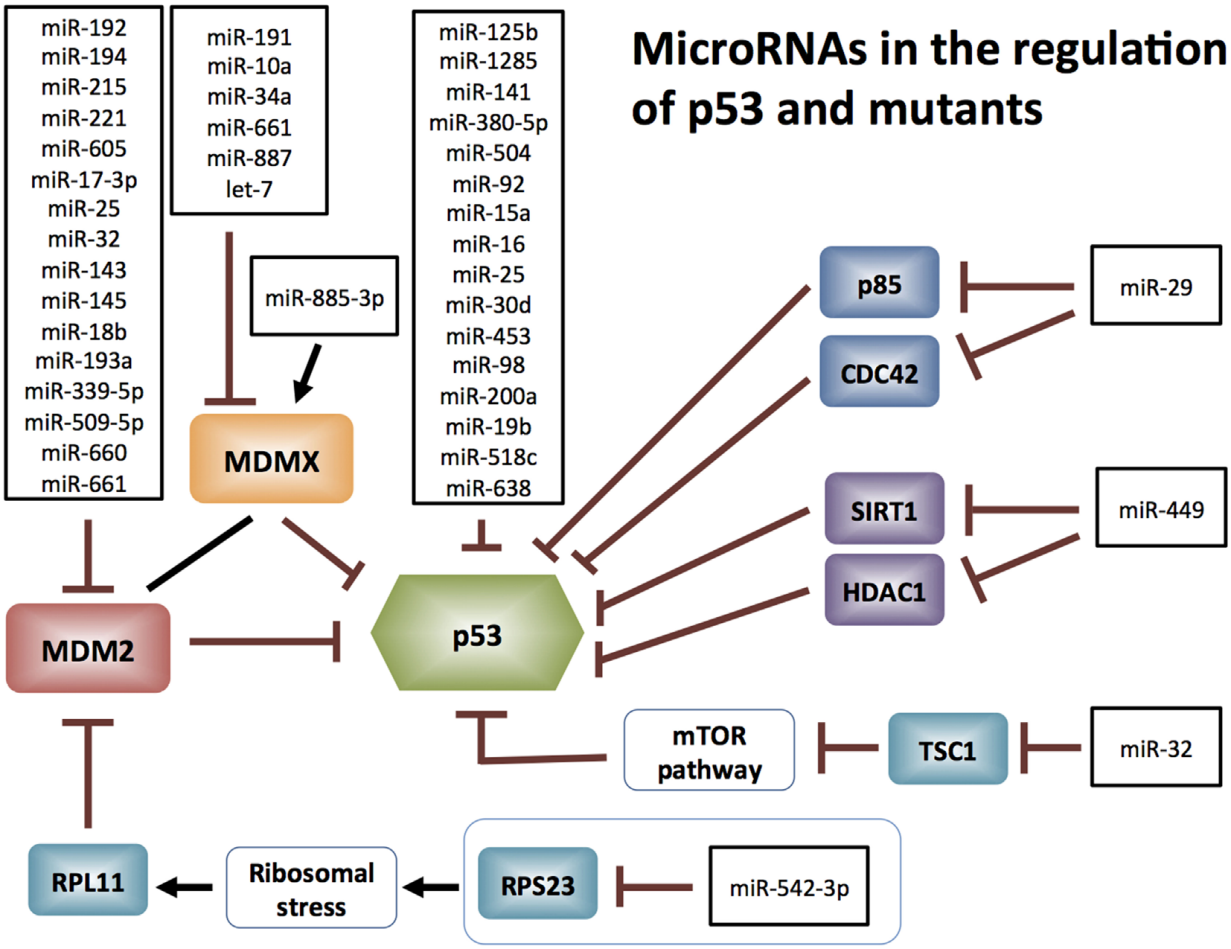
**MicroRNAs targeting p53: miR-125b, miR-504, miR-1285, miR-92, miR-141, miR-380-5p, miR-15a, miR-16, miR-25, miR-30d, miR-200a [reviewed in Ref. (**88)**], miR-453 (**89)**, miR-98 (**89)**, miR-19b (**90)**, miR-518c (**91)**, and miR-638 (**91). MicroRNAs targeting MDM2: miR-192, miR-194, miR-215, miR-221, miR-605, miR-17-3p, miR-193a, miR-25, miR-32, miR-143, miR-145, miR-18b, miR-661 [reviewed in Ref. (15)], miR-339-5p (92, 93), miR-660 (94), and miR-509-5p (95). MicroRNAs targeting MDM4: miR-191, miR-10a, miR-885-3p, miR-34a, miR-661 [reviewed in Ref. (15)], miR-887 (96), and let-7 (11).

The expression of both wt and mutant p53 are subject to miRNA regulation directly. MiRNAs targeting *p53* mRNA are incapable of discriminating between its wt and mutant mRNAs, unless an miRNA directly targets a mutated site. MiR-125b was the first miRNA demonstrated to bind *p53* 3′-UTR mRNA causing down-regulation of p53 protein and a consequent reduction in its activity, in human neuroblastoma cells and primary human lung fibroblasts (14). Additional p53-directed miRNAs have been identified both experimentally and from *in silico* analyses (Figure 1).

MicroRNAs also regulate p53 protein stability indirectly, by targeting its key regulators. For instance, the E3 ubiquitin ligase Mdm2, which is the major negative regulator of p53 [reviewed in Ref. (8, 15)] is extensively targeted by miRNAs for degradation. As a consequence of Mdm2 targeting, p53 (wt or mutant) is released from Mdm2-mediated ubiquitination and subsequent proteasomal degradation (Figure 1). Mdm4 (also known as MdmX), which is an Mdm2-related protein, is another key inhibitor of p53 transcriptional activity (16, 17) and is also targeted by miRNAs (Figure 1). Although miRNAs predominantly degrade mRNAs, an exceptional instance is miR-885-3p, which engages the 5′-UTR of Mdm4 mRNA and elevates Mdm4 protein levels (12).

Apart from Mdm2 and Mdm4, miRNAs also stabilize p53 through other regulatory pathways (Figure 1). MiR-29 activates p53 by targeting p85-alpha and CDC42 (18), miR-449 targets SIRT1 and HDAC1 (19), and miR-32 targets TSC1 and activates mTOR in human glioblastoma multiforme (20), all of which lead to the stabilization of p53. Recent study by Wang et al. demonstrated that miR 542-3p directly targets RPS23, resulting in subsequent RPL11 up-regulation, inhibiting Mdm2 and ultimately reducing proteasomal degradation of p53 (21).

Significantly for cancer, interaction of miRNAs with wt and mutant p53 is not unidirectional, and expression levels and biogenesis of miRNAs are affected by p53. Suzuki et al. demonstrated that wt p53 enhances post-transcriptional maturation of miR-16-1, miR-143, and miR-145 in response to DNA damage, while mutant p53 attenuates miRNA processing (22). Muller et al. further demonstrated that mutant p53 modulates miRNA ­processing, through direct inhibition of TAp63-mediated transcriptional activation of Dicer, and also through a TAp63-independent manner (23). Apart from global modulation of miRNA biogenesis, mutant p53 also affects expression of miRNAs, principally by downregulating tumor-suppressive miRNAs – miR-130b in endometrial cancer (24), miR-27a in breast cancer cells (MDA-MB-468) (25), miR-223 in breast and colon cells (26), let-7i in breast cancer and DLD1 cells (colorectal cancer) (27), and miR-205 (28), and elevating oncogenic miRNAs: miR-128-2 (29) and miR-155 in breast cancer cells (30) to mediate its oncogenic functions.

These studies collectively suggest that intertwined regulation of miRNAs, wt, and mutant p53 is vital to cancer. Given the importance of ncRNA in the regulation of wt and mutant p53, ncRNA represents feasible therapeutic targets for the development of new approach targeting mutant p53 in cancer.

## Regulation of Mutant p53 Protein Stability

Overall, wt p53 is predominantly regulated at the protein stability level, under normal and stress conditions. Extensive study has defined that p53 stability is dictated by a variety of stabilizing post-translational modifications (PTM), while its degradation is largely the consequence of ubiquitination, executed by several E3 ligases [reviewed in Ref. (31, 32)]. Despite a drastic difference between the stability of mutant versus wt p53 in cancer cells, the majority of the regulatory pathways of p53 are shared between wt and mutant p53. However, a number of key differences promote the chronic stabilization and activation of mutant p53, which drive its oncogenic GOF. Understanding the regulation of mutant p53 has direct clinical implications. In this section, we will cover the major levels of mutant p53 regulation, with a focus on the degradation of mutant p53.

## Regulation of Mutant p53 Degradation

p53 stability is tightly controlled by ubiquitin E3 ligases, which together with the enzymatic activities of E1 and E2, and in certain cases also E4 ligases coordinate the efficient degradation of proteins through the 26S proteasome machinery (33). The temporal and spatial modulation of the degradation of p53 is achieved by PTMs described below. In this section, we will outline the major E3 ligases that have been shown to control mutant p53 stability. A paradigm shift in our understanding of mutant p53 stability was demonstrated in the studies of mutant p53 knock-in mice (5, 34). These papers showed for the first time that mutant p53 is inherently labile *in vivo*.

**Figure 2:**
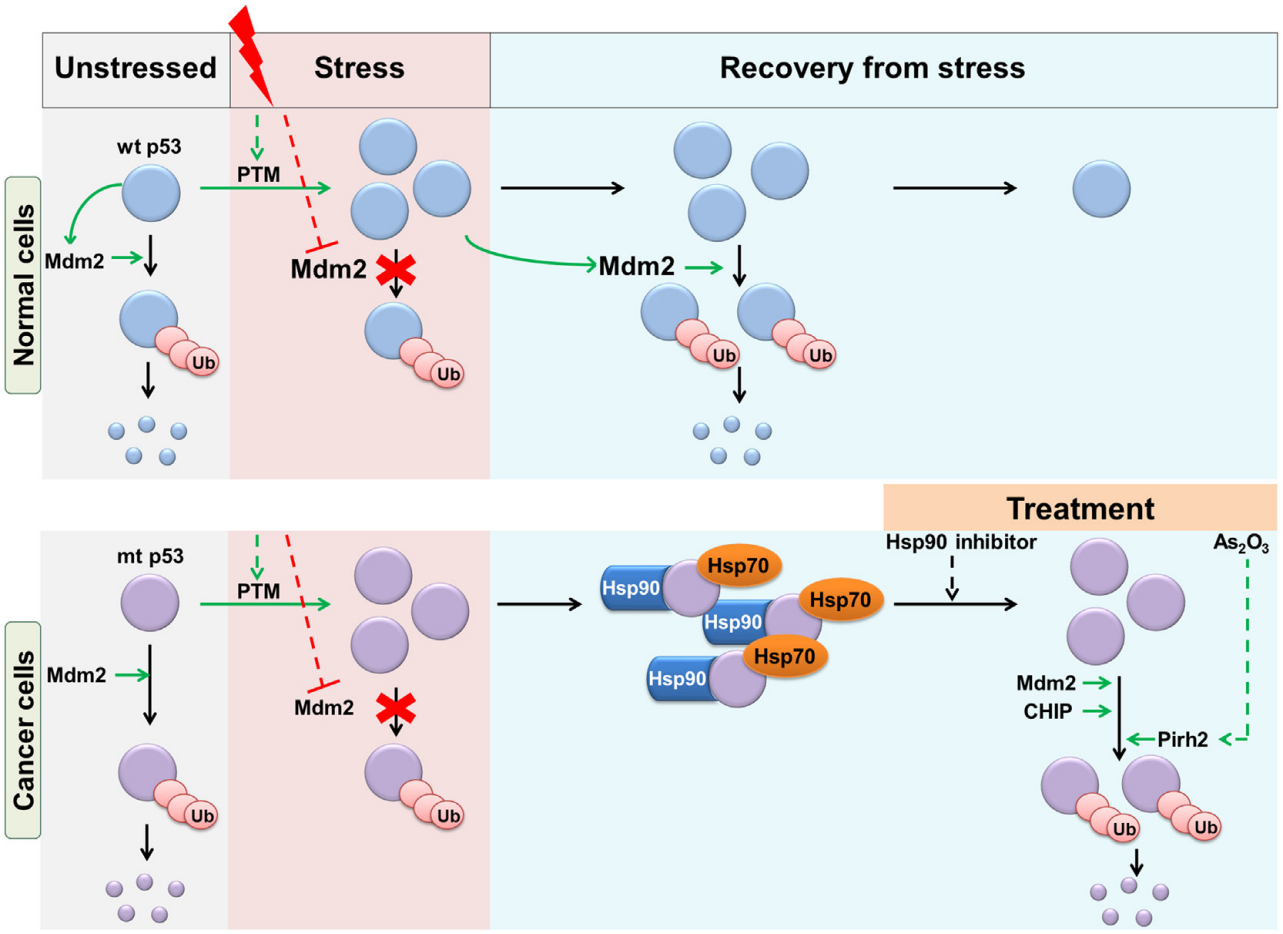
**Regulation of wt and mutant p53 stability in response to stress**. Wt and mutant p53 are maintained at basal levels by Mdm2-mediated degradation. Upon stress stimulus, events such as post-translational modifications (PTM) result in stabilization and accumulation of p53. During recovery from stress, wt p53 returns to basal levels as a result of negative feedback through Mdm2. However, mutant p53 accumulates due to its failure to transactivate Mdm2. Mutant p53 is also protected from Mdm2 and CHIP mediated ubiquitination by chaperone proteins. This can be overcome by treatment with Hsp90 inhibitors. Treatment of cells with As_2_O_3_ induces the E3 ligase Pirh2, which ubiquitinates mutant p53.

In the case of wt p53, the key physiological E3 ligase is Mdm2, which maintains p53 at low levels under basal conditions, and during recovery from stress (35, 36). Similarly, Mdm2 maintains the low basal levels of mutant p53 *in vivo* (7). In contrast to wt p53, mutant p53 does not form a feedback loop with Mdm2, as it is incapable of inducing Mdm2 transcription (37). Therefore, following stress-induced stabilization of wt and mutant p53, only wt p53 recovers to basal levels under the influence of Mdm2 (Figure 2). This can be corrected by enforced expression of Mdm2, which is able to efficiently degrade of mutant p53 (35). Mdm2 interacts with multiple domains of p53, which allows it to bind even to conformational p53 mutants, which are missense p53 mutations that either locally or globally disrupt the structure of p53, as distinct from “DNA contact mutants” (38, 39). The overall efficiency of mutant p53 ubiquitination, however, is reduced compared with that of wt p53 (40). The ubiquitination of mutant p53 is also enhanced by the activity of other E3 ligases: CHIP and Cop1 (40). Although ubiquitination of p53 by Mdm2 is enhanced by ubiquitin-interacting protein, hHR23a, the consequent ubiquitinated p53 accumulates but is not degraded (41). In a series of key *in vivo* studies, Terzian et al. (7) and Suh et al. (20) demonstrated that stress conditions (including oxidative stress, DNA damage) and oncogenic stress (such as the loss of p16) promote stabilization of mutant p53 and contribute to tumorigenesis. Subsequent studies have shown that multiple oncogenic effects can stabilize mutant p53 *in vivo* and drive its oncogenic functions. Interestingly, at least in the case of PML loss, the impact on mutant p53 GOF is gender-specific (42).

Degradation of mutant p53 has also been described to occur via alternative forms of autophagy. The first is: “Macroautophagy,” which is triggered in response to starvation to recycle cellular contents through the lysosomes. A specific form of starvation is glucose restriction, which increases mutant p53 deacetylation, and sends it to degrade through the autophagic machinery. This degradation is Mdm2-dependent (43), but does not involve the proteasome (43, 44). A second form is: selective “chaperone-mediated autophagy” (CMA), in which specific cytosolic proteins are engaged by heat shock proteins (HSPs) and targeted to lysosomes. CMA is a normal cellular process that becomes more active in response to nutrient deprivation (43–46). Although mechanisms like autophagy seem to be less specific than proteasomal degradation, it has been observed that ubiquitinated proteins are targeted for lysosomal degradation and could play a major role in regulating mutant p53 (47).

CHIP is a chaperone-dependent E3 ligase able to ubiquitinate misfolded proteins, a process which is assisted by the Hsp70/90 chaperone machinery. Hsp90-bound substrates are protected from ubiquitination, whereas Hsp70-bound substrates are ubiquitinated by CHIP (48). Interestingly, Hsp70 is found to partially inhibit Mdm2-mediated degradation of mutant p53. This apparent discrepancy in Hsp70 activity with respect to the two E3 ligases is not completely understood. In contrast, Hsp90 protects mutant p53 from both CHIP and Mdm2-mediated degradation (49, 50). This is because mutant p53, unlike wt p53, forms a stable complex with Hsp90 (51, 52). Inhibition of Hsp90 by 17AAG or siRNA against HSF1 (a major transcription factor for Hsp) enhances the ubiquitination and degradation of mutant p53 (Figure 2) (53, 54). Indeed, it was observed that inhibition of Hsp90 reduced the viability of mutant p53-expressing cancer cells of breast (MBA-MB-468, MDA-MB-231, T47D), prostate (DU145), and colon (SW480) (49). Similarly, inhibition of HDAC6, a positive regulator of Hsp90, destabilizes mutant p53 and is preferentially toxic to mutant p53-expressing cancer cell lines (54, 55). Hsp90 directly, or indirectly via its transcriptional activator HSF1, is upregulated in many cancer types, which may contribute to mutant p53 stabilization (56). Mutant p53 exists in a positive forward loop with HSF1. Mutant p53 enhances HSF1 recruitment to DNA, thereby increasing the levels of HSPs, which further stabilize mutant p53 (57, 58). Indeed, treatment with Hsp90 inhibitor, 17DMAG (a derivative of 17AAG), can greatly reduce lymphoma formation and is known to improve survival in mice with mutant p53 (59). Lastly, RING domain containing E3 ligase, Pirh2, which directly ubiquitinates wt p53 (60), also interacts with and promotes the ubiquitination of mutant p53 (61, 62). Since Pirh2 is a p53 target gene, this negative feedback regulatory loop is interrupted by mutations in p53 (60). Treatment of mutant p53-expressing cells with Arsenic trioxide can induce Pirh2-mediated proteasomal degradation of mutant p53 (Figure 2) (62). The role of other E3 ligases, including ARF-BP1, which regulate wt p53, have been shown not to regulate mutant p53 [(40) and reviewed in Ref. (8)].

Countering the E3 ligases are the deubiquitinating enzymes (DUBs), which cleave ubiquitin from proteins. Unlike E3 ligases, the role of DUBs in controlling mutant p53 stability is poorly explored. USP10 deubiquitinates and stabilizes both wt and mutant p53 (63). Inhibition of USP10 by the protein spautin-1 reduces mutant p53 levels under glucose-restricted conditions (46). The USP7 DUB has a complex interplay with both wt p53 and Mdm2, and it deubiquitinates the p53 activator, Tip60 (64). To date, no correlation between USP7 and p53 status has been identified in cancers (65, 66). In wt p53-expressing cell lines, inhibition of USP7 stabilizes p53 and promotes apoptosis. But in at least one mutant p53-expressing cell line, the inhibitor had no effect (67). ABRO1 is able to deubiquitinate wt p53 and stabilize it by facilitating its interaction with USP7. However, there is no information pertaining to its ability to deubiquitinate mutant p53. Overexpression of ABRO1 in HT29 (colorectal adenocarcinoma) and BT474 (breast cancer) cells (both expressing mutant p53) results in increased cell growth, which can be correlated with mutant p53 stability (68). Another DUB, UCHL1, stabilizes wt and mutant p53 levels in breast cancer cell lines and affects cell viability by a mechanism remaining to be explored (69). USP29 can also deubiquitinate p53 in response to oxidative stress (70).

Although much of the focus in the field has been devoted to the 26S-mediated proteasomal degradation of p53 during post-stress recovery, the 20S proteasome has been identified as the destination of unmodified p53 that is inherently unstable, unless protected by the NADH quinone oxidoreductase 1 (NQO1). Specifically, inhibition of NQO1 (for example by dicoumarol) promotes p53 degradation through the 20S proteasome in an Mdm2-independent manner. Interestingly, mutant p53 interacts strongly with NQO1, rendering it resistant to NQO1 inhibitors [reviewed in Ref. (71)]. Pertinently, NQO1 is elevated in many cancers, which may contribute to stabilization of mutant p53 in these cases [reviewed in Ref. (72)].

Free ribosomal proteins are known to regulate the Mdm2/MdmX-p53 axis and activate wt p53, thereby inhibiting tumor proliferation [reviewed in Ref. (73)]. For example, RPS27 is repressed by wt but not mutant p53, and increased expression of RPS27 stabilizes mutant p53 protein, thereby forming a feed forward loop in cancer (74). On the other hand, RPL26 not only binds to the 5′-UTR of p53 mRNA and enhances translation but also interacts with Mdm2 and protects p53 from degradation (75, 76).

## Post-Translational Modifications of Mutant p53

The regulation of wt p53 degradation is modulated by PTMs. Wt p53 is extensively modified post-translationally in response to stress conditions, which lead to the stabilization and/or activation of p53 [reviewed in Ref. (31)]. Critically, in most of the tested cases, the PTMs of p53 are non-discriminatory between wt and mutant proteins (7, 31, 77). Some of these modifications contribute to mutant p53 stability by shielding it from degradation by Mdm2 (Figure 2). Specifically, phosphorylation of p53 on serine 20 and threonine 18 in response to DNA damage protects it from Mdm2 and leads to its activation and stabilization (77–79). These phosphorylations are also induced on mutant p53 in response to stress (49, 77). Mutant p53 also escapes from Mdm2 by constitutive phosphorylation by ERK (80). Similarly, activation of SIRT1, which deacetylates mutant p53, can reduce mutant p53 levels in triple-negative breast cancer cell lines, revealing the role of acetylation in stability of the mutant protein (81, 82).

In addition to mutant p53 itself, modifications of Mdm2/MdmX contribute to the protection of mutant p53 from these key inhibitors. In response to DNA damage, Mdm2 is phosphorylated by ATM and c-Abl, which compromises the ability of Mdm2 to degrade p53 (83, 84). ATM-mediated phosphorylation contributes also through the impaired oligomerization of Mdm2 (85). Similarly, MdmX, the key inhibitor of p53 is phosphorylated by ATM and c-Abl, which impairs its capacity to inhibit p53 (86, 87). To what extent these key phosphorylations affect mutant p53 is yet to be demonstrated. The indiscriminate modifications of wt versus mutant p53 in response to stress can contribute to mutant p53 accumulation and activation.

## Concluding Remarks

While wt and mutant p53 have distinct and opposing effects on cancer cells, many aspects of their regulation are shared. The majority of the positive and negative regulators of wt p53 that have been tested have a similar regulatory effect on mutant p53. Critically, however, the tightly controlled myriad of positive and negative auto-regulatory loops, which govern wt p53 levels, is uncoupled in the context of mutant p53. In addition, mutant p53 is recognized as a misfolded protein by the heat shock protein chaperons. Together, these contribute to the protection of mutant p53 from the well-coordinated recovery from stress conditions. This results in the chronic accumulation of active mutant p53, which exerts its gain of functions. It is therefore of prime importance to screen patients for p53 mutations prior to treatments, which are known to activate and stabilize p53. The identification of mechanisms that protect mutant p53, as shown by the chaperon HSP proteins, identifies novel approaches to expose mutant p53 to its negative regulators and drive its destruction. Future studies identifying the unique protectors of mutant p53 are a rational approach to define novel approaches to target mutant p53 in cancer cells.

## Author Contributions

RV contributed to writing and editing the paper and prepared a figure. KT contributed to writing and prepared a figure. PJM contributed to writing. SH and YH contributed to writing and editing the paper.

## Conflict of Interest Statement

The authors declare that the research was conducted in the absence of any commercial or financial relationships that could be construed as a potential conflict of interest.
